# Nose to tail, roots to shoots: spatial descriptors for phenotypic diversity in the Biological Spatial Ontology

**DOI:** 10.1186/2041-1480-5-34

**Published:** 2014-08-11

**Authors:** Wasila M Dahdul, Hong Cui, Paula M Mabee, Christopher J Mungall, David Osumi-Sutherland, Ramona L Walls, Melissa A Haendel

**Affiliations:** 1 Department of Biology, University of South Dakota, Vermillion, SD, USA; 2 National Evolutionary Synthesis Center, Durham, NC, USA; 3 School of Information Resource and Library Science, University of Arizona, Tucson, AZ, USA; 4 Lawrence Berkeley National Laboratory, Berkeley, CA, USA; 5 Department of Genetics, University of Cambridge, Cambridge, UK; 6 The iPlant Collaborative, Bio5 Institute, University of Arizona, Tucson, AZ, USA; 7 Library and Department of Medical Informatics & Epidemiology, Oregon Health & Science University, Portland, OR, USA

**Keywords:** Anatomy, Spatial relationships, Position, Axes, Reasoning, BSPO, Ontology, Phenotype

## Abstract

**Background:**

Spatial terminology is used in anatomy to indicate precise, relative positions of structures in an organism. While these terms are often standardized within specific fields of biology, they can differ dramatically across taxa. Such differences in usage can impair our ability to unambiguously refer to anatomical position when comparing anatomy or phenotypes across species. We developed the Biological Spatial Ontology (BSPO) to standardize the description of spatial and topological relationships across taxa to enable the discovery of comparable phenotypes.

**Results:**

BSPO currently contains 146 classes and 58 relations representing anatomical axes, gradients, regions, planes, sides, and surfaces. These concepts can be used at multiple biological scales and in a diversity of taxa, including plants, animals and fungi. The BSPO is used to provide a source of anatomical location descriptors for logically defining anatomical entity classes in anatomy ontologies. Spatial reasoning is further enhanced in anatomy ontologies by integrating spatial relations such as *dorsal_to* into class descriptions (e.g., ‘dorsolateral placode’ *dorsal_to* some ‘epibranchial placode’).

**Conclusions:**

The BSPO is currently used by projects that require standardized anatomical descriptors for phenotype annotation and ontology integration across a diversity of taxa. Anatomical location classes are also useful for describing phenotypic differences, such as morphological variation in position of structures resulting from evolution within and across species.

## Background

**Figure 1:**
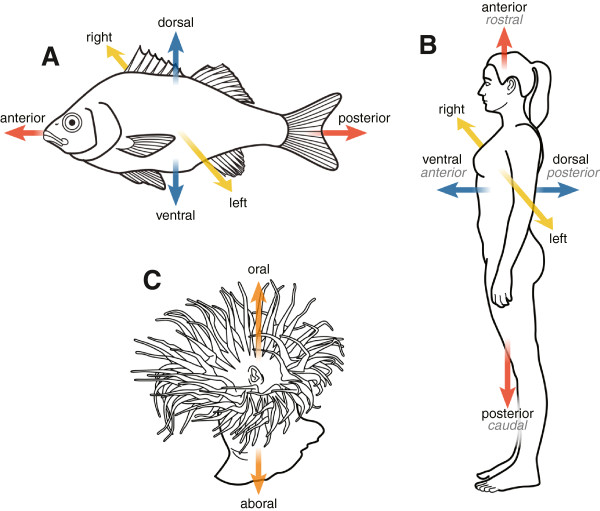
**Comparison of primary organismal axes designated in a diversity of species and their representation in BSPO.** In fishes **(A)** and in humans **(B)**, ‘anterior-posterior axis’ (narrow synonym ‘rostral-caudal axis’ in humans) is shown in red, ‘dorsal-ventral axis’ (narrow synonym ‘anterior-posterior axis’ in humans) shown in blue, and ‘left-right axis’ shown in yellow. A cnidarian (sea anemone) **(C)** is bilaterally symmetrical and has an ‘oral-aboral axis’, shown in orange.

Variation among anatomical phenotypes, whether across species or between mutant and wildtype model organisms, frequently involves changes in position and orientation of structures. Among fish species, for example, the position of the mouth may be ventral, dorsal, or terminal; bony vertebral processes may be oriented laterally or medially; pelvic fins may be located posteriorly or anteriorly relative to the abdomen. Computation across phenotypes thus requires a vocabulary of positional terms to understand the patterns of variation in the positioning of structures relative to others within and between organisms, and to understand the possible relationships to gene expression and regulation. Positional terms have long been used in anatomy to describe the spatial aspects of the impressive diversity of organismal forms of both plants and animals. For example, positions in animals are often described in relation to those of a bilaterally symmetrical animal (Figure 1). Accordingly, the primary or main axis is considered the anterior-posterior (AP) axis, which extends longitudinally from head to tail. The dorsal-ventral (DV) axis is recognized in that ventral typically faces toward, and dorsal away, from a substrate (meaning towards the ground for land-dwelling organisms or towards the ocean or river/lake bottom for marine or aquatic organisms), whereas the left-right (LR) axis is defined in relation to a plane running along the anterior-posterior midline. We created the Biological Spatial Ontology (BSPO) to develop, define, and standardize terms that can be used to describe spatial and topological relationships, at multiple biological scales from cells to whole organisms, and across diverse taxa.

In the past two decades the developmental and genetic underpinnings of positional axes have been investigated for model species, and highly conserved key patterning molecules have been identified across widely divergent taxa. Overlapping patterns of Hox gene expression, for example, are required for organization along the AP body axis in bilaterian animals [1]. Wnt/β-catenin expression has also been shown to determine primary body axis orientation in both bilaterian and non-bilaterian animals [2]. The DV axis is patterned by the chordin–bone morphogenetic protein (BMP) network and is conserved across organisms as diverse as flies and humans (reviewed in [3]). Nodal signaling has been shown to control LR symmetry, which also appears to have an ancient prebilaterian origin [4]. Within plants, homeobox genes, such as knotted-like homeobox (*knox*), also play a central role in spatial developmental patterns [5]. Although specification of organismal axes may appear straightforward with respect to their application within model organisms (e.g., *Arabidopsis, Caenorhabdites elegans, Drosophila*, *Danio rerio*, *Xenopus*, mouse, etc.), there are taxon-specific differences in the application of spatial terms, such as to human anatomy, that render the development of a universal terminology complicated. Moreover, there are fundamental differences across the more than 35 animal body plans (e.g., tapeworms, sea urchins) and various plant growth forms (e.g., tree, shrub, herb, and thallus) that present some very difficult axes to interpret. Despite these challenges, which we describe further below, the development of a set of spatial classes is necessary for query and description of phenotypes across species.

Here we describe the development of the BSPO, which contains 146 classes and 58 relations representing anatomical axes, gradients, regions, sections, sides, and surfaces that apply to whole organisms and their parts. The BSPO is integrated with other ontologies and is currently used by projects that require standardized spatial descriptors for anatomy ontologies, ontology integration, and phenotype annotation. For example, the free-text description “anterodorsal margin of opercle” can be represented formally as BSPO:‘anterodorsal margin’ *part_of* some ‘opercle’ (the latter class from an anatomy ontology). Because it is driven by research needs, the spatial terminology currently represented in BSPO is particularly developed for animals and, to a lesser extent, plants. However, BSPO is organized in a framework that is flexible enough to incorporate spatial terminology for other taxa (e.g., fungi).

## Results and discussion

### Ontology organization and content

**Figure 2:**
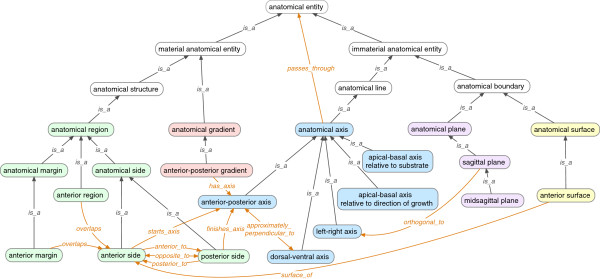
**Organization of high-level spatial classes in BSPO and some of their children.** ‘Anatomical region’ (green fill), ‘anatomical gradient’ (pink fill), ‘anatomical axis’ (blue fill), ‘anatomical plane’ (purple fill), and ‘anatomical surface’ (yellow fill). Parent classes from CARO are shown with white fill. Subclass (*is_a*) relations are shown in black and spatial relations in orange.

Classes^a^ in BSPO represent various aspects of spatial organization and are partitioned into categories for anatomical axes (14 classes), anatomical surfaces (12 classes), anatomical regions (81 classes, including margins and sides), anatomical gradients (6 classes), and anatomical planes (7 classes) (Figure 2). BSPO classes for ‘anatomical compartment’ and ‘anatomical compartment boundary’ (11 classes) refer to anatomical structures defined by lineage restriction [6] rather than by axial position, and thus these classes will be moved to the Common Anatomy Reference Ontology (CARO) [7] in the future. Individual compartments and their boundaries are typically named with respect to some axis. For example, most of the imaginal discs and embryonic segments of insects are bisected by a boundary running medial to lateral that cells do not cross during development [8]. The regions of the disc or segment anterior and posterior to the boundary are referred to as anterior and posterior compartments, respectively, while the boundary is referred to as the anterior-posterior compartment boundary. BSPO provides relationships that allow these compartments and boundaries to be defined with respect to anatomical axes, in individual cases, but general classes such as ‘anterior compartment’ seem of dubious usefulness and so will not be maintained in either BSPO or CARO.

BSPO classes are linked by a rich set of 58 relationship types. In addition to their logical relationships, all BSPO classes have text definitions that are written as broadly as possible to encompass taxonomic variability in body form. Synonyms are included where applicable and include commonly used abbreviations for terms such as “LR axis” for ‘left-right axis’.

BSPO is open to all users and freely available in OBO and OWL formats at http://purl.obolibrary.org/obo/bspo.{ obo,owl }. BSPO can also be browsed online at http://www.ontobee.org/browser/index.php?o=BSPO.

### Anatomical axes

#### Primary organismal axes

Axes form the basis of the BSPO, with other concepts, such as relations and planes, defined in terms of these axes. In animals, three whole body axes are generally applicable (described below). Unlike the case in animals, there is generally no single primary organismal axis for a whole plant. Instead, axes are described for one or more modular organs that compose a plant, such as shoots (stems and branches), roots, and phyllomes (leaves, petals, etc.) (see “Axes of organism parts”, below).

In animals, the AP, DV, and LR axes (Figures 1A, B) are applicable to Bilateria and most of their descendants. The Bilateria include all metazoans except the sponges (Porifera), placozoans, cnidarians, and ctenophores. Most sponges are asymmetrical as adults, although an AP axis has been identified in their larvae [9]. Cnidarians are primitively bilaterally symmetrical [10], with radial or biradially symmetrical axes developing in more derived members of the clade (Figure 1C). Genes regulating bilaterian head development are expressed in the sea anemone at the larval aboral pole, indicating that the anterior, head-forming, region of bilaterians and the aboral region of cnidarians may have been derived from the same domain of their last common ancestor [11]. Bilateral symmetry of the body, including an anterior head with an oral opening and a posteriorly extended trunk/tail with an anal opening, is thought to characterize the common ancestor of Bilateria. Many textbook definitions of the three fundamental axes of bilaterians (AP, DV, LR) reference structures such as “head”, “oral opening/mouth”, “anus”, ”tail” or “gut” that are not present in all larval or adult bilaterians. Our definitions for these axes also reference anatomical structures, but aim to use only the minimum that are those hypothesized to be present based on phylogenetic reconstruction of the ancestral bilaterian [12].

**Figure 3:**
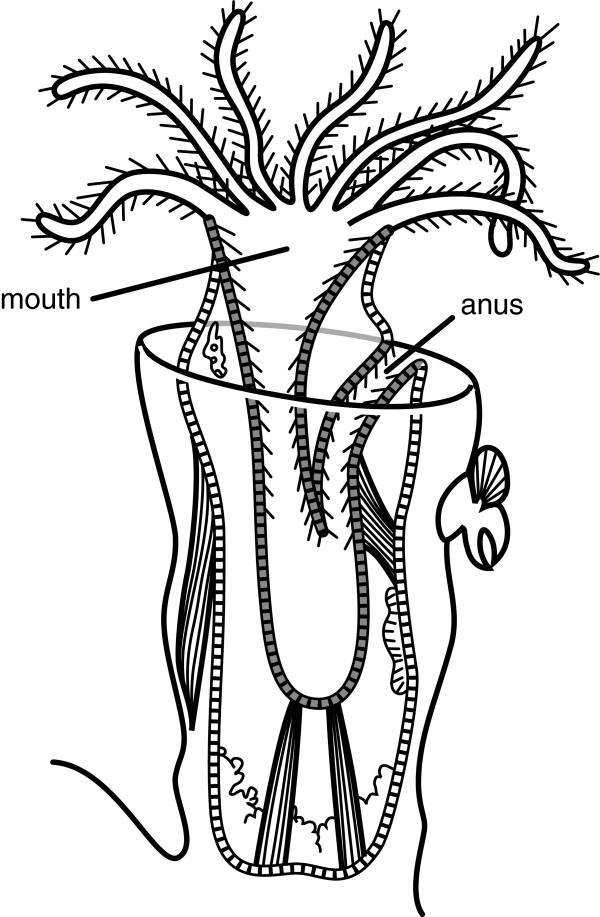
**An individual zooid of the colonial ectoproct *Bugula.*** This species possesses a U-shaped gut and the location of the anus is adjacent to the mouth. Image based on illustration from the BIODIDAC image library.

In defining ‘anterior-posterior axis’ in BSPO, we designate “anterior” as the end of the animal with a “head”. Interestingly, a head, however defined (e.g., based on concentration of neurons [11], sensory structures, oral opening), has been lost multiple times in development and evolution (e.g., adult tunicates, echinoderms, bivalve molluscs, ectoprocts, endoprocts), and as such the AP axis is hard or impossible to identify in these taxa. Although the oral opening/mouth is used as a proxy for an anterior end, it, as well, has been lost multiple times in various taxa (acanthocephalans, pogonophorans) or moved posteriorly in others (flatworms such as planarians) [13]. On the other end, criteria for recognizing “posterior” are conventionally related to an anal opening at or near to the terminus of the body. However, given multiple independent losses of an anal opening (e.g., gnathostomulids, some echinoderms) and the many taxa with a U-shaped gut in which the anus is adjacent to the mouth (e.g., sipunculids, ectoprocts, entoprocts, some gastropods) (Figure 3), using a digestive tract as a proxy for the longitudinal axis (AP) of the body is problematic. Interestingly, the U-shaped gut in some taxa is AP regionalized using highly conserved transcription factors [14]. As Minelli [15] points out, many taxa, such as those with a U-shaped gut, demonstrate dissociation between an apparently evident elongate AP somatic body axis and a very different visceral axis. In fact, dramatic metamorphic development of many invertebrates renders body axes very difficult to interpret (e.g., [16]). Even in taxa with distinct head and tail ends, modifications in body form can result in unconventional application of AP axis terminology. For example, seahorses, with their distinct “upright” posture, orient their AP axis perpendicular relative to the substrate rather than parallel [17]. Differential application of axes to the body and its parts is necessary in cases where they have been dissociated in development or evolution.

Developmental and evolutionary changes to the DV axis likewise pose challenges for simple application of terminology. In BSPO, ‘dorsal-ventral axis’ is defined as “An axis that is approximately perpendicular to the anterior-posterior axis and that extends through the horizontal plane of the body”. An inversion of the DV axis occurred during evolution resulting in correspondence between the ventral side of arthropods and the dorsal side of vertebrates, as evidenced by phenotype (position of the neural cord/tube) and inversion of the Chordin/BMP/Tolloid pathway markers [18].Another challenge in the application of the terminology of the fundamental bilaterian axes (AP, DV) is that these axes are uniquely conflated in humans and other anthropoid apes that are bipedal. In humans, “superior” is applied to the head end (anterior) and “inferior” towards the feet. “Anterior” and “posterior” are applied to the human front (ventral) and back (dorsal), respectively (see also Axes of organism parts, below). We have added human-specific terminology as synonyms to BSPO (Figure 1B) to assist in unambiguous reference to anatomical position when comparing anatomy or phenotypes across species.

In BSPO, the ‘left-right axis’ is defined as “An axis that extends through an organism from left to right sides of body, through a sagittal plane”, and it is *orthogonal_to* ‘sagittal plane’. The LR axis of many organisms is also modified in development and evolution. For example, flatfishes (order Pleuronectiformes) undergo a dramatic developmental change in the LR body axis. In ontogeny, the left or right side of the body comes in contact with the substrate, and one eye migrates to the other half of the head. Thus one side (left or right depending on the species) has two eyes and the side in contact with the substrate is referred to as "eyeless" or "blind". Description of this modified anatomy requires specialized terms to refer to the “blind side” and “eyed side” of the fish. Other structures typically located along the midsagittal plane, such as the dorsal fin, are displaced horizontally. Reasoning across flatfish and unmodified vertebrate eye morphologies may thus require specifying the spatial location (left or right side) of the blind or eyed side of the organism.

Despite the modifications to the primary axes we describe above, larvae and adults of many animal taxa do in fact retain the ancestral bilaterian AP, DV, and LR axes, and many conserved molecular and genetic determinants of these axes have been described in model organisms. Few of the non-model taxa with the interesting deviations from symmetry described above have been investigated from a developmental or genetic standpoint, and thus much remains to be discovered and understood about axis specification.Several other primary organism axes are represented in BSPO. The ‘medial-external axis’ extends from an internal point towards the outside of the body or body part. This class is a superclass of ‘medial-lateral axis’ (ML) and ‘medial-radial axis’ (MR). In animals, the ML axis applies to the left or right sides of a bilaterally symmetrical animal. The ‘oral-aboral axis’ (Figure 1C) is defined as the axis that extends from the oral opening to the furthest point in an organism that is directly opposite. It is the major axis in cnidarians, ctenophores, and echinoderms. During development, an ‘animal-vegetal axis’ (AV) is defined for most animal eggs, where the yolky (less rapidly dividing) end is “vegetal” and the less yolky (more rapidly dividing) end is “animal”. These terms are also often applied to the poles (e.g., “the animal pole”) and the hemispheres (e.g., “the animal hemisphere”).

#### Axes of organism parts

**Figure 4:**
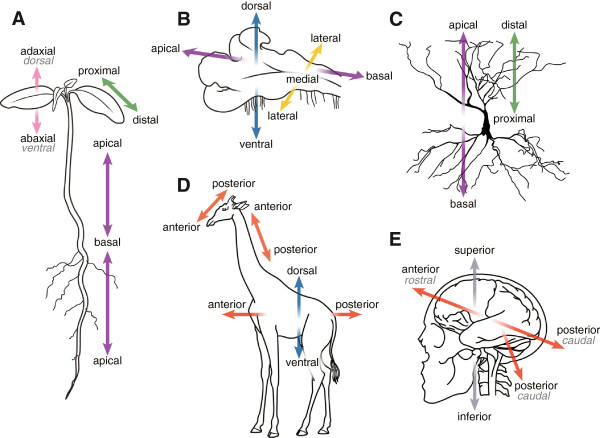
**Axes applied to organism parts.** In vascular **(A)** and non-vascular plants **(B)**, the ‘apical-basal axis relative to direction of growth’ (purple) runs in the direction of apical growth, in both shoots and roots. For lateral organs such as branches or leaves **(A)**, the primary axis is the ‘proximal-distal axis’ (green) and the ‘adaxial-abaxial axis’ (pink). In plants or organisms with a thalloid growth form **(B)**, the ‘apical-basal axis relative to direction of growth’ often runs parallel to the substrate, resulting in a ‘dorsal-ventral axis’ that runs perpendicular to the substrate and a ‘medial-lateral axis’ that is perpendicular to the ‘apical-basal axis’. **C)** Hippocampal pyramidal neuron, showing the application of the BSPO classes ‘apical-basal axis relative to substrate’ and ‘proximal-distal axis’ to the whole cell or portions thereof. **D)** AP axes for the head, neck and trunk of the giraffe. Note that these axis definitions delineate a “bent” version of the primary AP axis. **E)** AP axis of the human brain (double-headed red arrow) relative to the AP axis of the body (single red arrow). Note the use of “superior” and “inferior” to refer to structures relative to the substrate.

In plants, as mentioned above, the axes primarily relate to organismal parts and are generally defined relative to the direction of growth. The main axis of growth is typically the apical-basal (AB) axis, which is determined by the growth of an apical meristem or apical cell. In BSPO, we refer to this axis as ‘apical-basal axis relative to direction of growth’ to distinguish it from the ‘apical-basal axis relative to substrate’ (described below), which is applied to animal bodies. In Figure 4A, which shows a seedling of a vascular plant, an ‘AB axis’ suggests a single straight line running through the center of the plant, from the tip of the root to the tip of the shoot apical meristem. However, even in this very simple plant, there are two AB axes, one for the shoot system and one for the root. Most plants have more complex, branching growth forms with multiple AB axes. While one might describe the abstract, overall shape of a plant (e.g., an ellipsoid, cube, or pyramid) and define axes for that shape, those axes would not necessarily relate to the actual axes along which the plant develops. The AB axis for a whole plant can be used only with the simplest of growth forms, such as a non-branching liverwort or fern thallus. In plants with secondary growth (that is, growth that thickens axial organs such as stems), the medial-radial (MR) axis extends from the center of the organ to the outside. The DV and ML axes for a whole plant are also used with thalloid growth forms (whether branching or not), because they grow roughly in a plane along the surface of the substrate (Figure 4B).

In animals, the ‘apical-basal axis relative to substrate’ is often applied to substrate-bound organisms such as Porifera, where the basal direction is towards the substrate. For bilaterian animals, this axis often refers to cell or tissue-level axes where one portion of the cell or tissue is adjacent to a substrate, such as a basal lamina or lamina propria, and the apical portion faces a lumen, for example an intestinal epithelial cell with its microvilli facing the lumen of the intestine.

In animals and plants, a ‘proximal-distal axis’ (PD) is used to describe the position of parts in relation to attachment to another part, such that parts closer to the plane of attachment (e.g., the point where a leaf attaches to a branch) are proximal and those further away are distal (Figure 4A). In animals, the terms “proximal” and “distal” are often applied to outgrowths of the body, such as limbs and other appendages such as antennae, parapodia, and feathers. In animals the regulatory gene *distal-less* has a role in specifying the PD axis, and it is expressed in the distal portion of many appendages [19]. Proximal-distal terminology can also be applied across different levels of anatomical organization to organs, tissues, and cells; for example, the “proximal/distal epiphysis of femur”, the “proximal/distal collecting tubule” of the kidney, or the “distal/proximal apical dendrite” of a neuron (Figure 4C).

In plants, “proximal” and “distal” should be applied to organs or organ parts that do not develop from an apical meristem (and therefore have no AB axis) such as vascular leaves, leaflets, petals, or sepals. The PD axis can also be used for organs with an apical meristem that branch from another organ, such as branches or lateral roots, but in these examples it is redundant with the AB axis. The ‘adaxial-abaxial axis’ (AA) is also important for leaves and other types of phyllomes, with adaxial being adjacent to the shoot axis (usually the top of the leaf) and abaxial being away from the shoot axis (usually the bottom of the leaf). If a leaf or other organ is held horizontally, the ‘adaxial-abaxial axis’ may be described as dorsal-ventral. The distribution of tissues varies along the AA axis in leaves, including characteristics of each surface, reflecting the different microclimates on the adaxial versus the abaxial sides of the leaf, such as sun exposure and humidity.

Medial-external axes are also applied to parts of an organism. The ‘medial-radial axis’ in plants is used to describe organs or organ parts that are roughly circular in cross-section, such as stems, roots, and petioles, while ‘medial-lateral axis’ is used to describe laminar (flattened) plant parts such as many leaves and petals or some shoot axes (e.g., cactus paddles) that expand through growth of marginal meristems [20]. Variation in the development of meristems along the AA, PD, or ML axes results in much of the variation found in leaf shapes. For example, some leaves that have a rounded cross-section, such as some species of *Sanseveria*, have an early developmental pattern in which growth of either the abaxial or adaxial leaf meristem is suppressed, to the effect that the opposite meristem (adaxial or abaxial, respectively) grows around to cover the entire surface of the leaf.

In cases where the application of axis terminology is difficult, molecular determinants may be used as evidence for spatial reference of body parts. For example, the region of the fin or limb bud in vertebrates with a high concentration of sonic hedgehog (Shh) is posterior because Shh “posteriorizes” the phenotype [21]. Note that this can apply to either portions of a given body axis, or to structures that are not themselves part of a main body axis (see also Anatomical gradients below).Axis terminology applied to substructures of an animal requires reference to a main axis of the body, such as “anterior” or “posterior”, and sometimes the ancestral condition of the body. For example, in the giraffe (Figure 4D), the AP axis is applied to several body segments (head, neck, trunk) and the DV axis is designated as perpendicular to the AP axis for each of these segments. As a result, the DV axis of the neck is nearly parallel to the AP axis of the trunk. Similarly, for humans and other bipedal anthropoids, the application of an organismal head or brain axis is uniquely conflated with the primary axis of the organism. In this case, the AP axis (often called rostral-caudal) of the human brain is at almost a right angle to the AP axis of the rest of the body (Figure 4E).

The traditional use of “superior” and “inferior” refers to parts that are the furthest or nearest to the substrate respectively. In BSPO, we define ‘inferior side’ and ‘superior side’ classes to support reference to the substrate. However, confusion can arise when these terms are applied to homologous structures across species where they may not retain the same relationship to the substrate. For example, the human superior vena cava is further from the substrate than the inferior vena cava, but in the mouse, these terms no longer reference differential distance from the substrate. For this reason, we do not recommend their use in defining axes or relations to axes, for structures that are likely to be compared across taxa.

The BSPO does not yet have a complete terminology for describing the spatial dimensions of fungal anatomy, which could be integrated with existing anatomy ontologies for fungi (Fungal Subcellular Ontology [22] and Fungal Anatomy Ontology (FAO; http://purl.obolibrary.org/obo/fao.owl)). Nonetheless, BSPO can easily accommodate the spatial terminology used to describe fungi, and some existing BSPO terms are applicable to fungal anatomy. For example, “lateral” is used in fungi, as in animals, to refer to the side of the organism [23], and the ‘medial-radial axis’ can be used to describe cylindrical structures in fungi such as the stem or stalk of a mushroom. The ‘dorsal-ventral axis’ and ‘medial-lateral axis’ used to describe thalloid plant structures (Figure 4B) could easily be applied to thalloid lichens. The terms “adaxial” and “abaxial” are used in fungi, similar to their application in plants, to describe the side of an anatomical structure that is adjacent to or away from the long axis of another structure. While the ‘abaxial-adaxial axis’ can be used fairly generally to describe multiple types of organs in plants, within fungi, “adaxial” and “abaxial” are restricted to describing the sides of basidiospores in relation to the basidium, a specialized cell or organ in the basidiomycetes [23].

#### Relations along anatomical axes

Fifty-eight relations have been specified for use with BPSO terms. For each axis in BSPO we define a pair of relations specifying relative position along the axis. For example, for the DV axis we have the relations *dorsal_to* and its inverse *ventral_to*. An entity x is *dorsal_to* an entity y if x is further along the DV axis than y towards the dorsum. Each of these relations is also declared to be transitive (i.e., if x is *dorsal_to* y, and y is *dorsal_to* z, then x is *dorsal_to* z). We also define non-transitive versions of these relations, e.g., *immediately_dorsal_to* and *immediately_ventral_to* as subproperties of the transitive forms. These are useful for specifying the order of serially arranged, contiguous structures such as the tagmata and segments of an arthropod body, the segments of an arthropod leg, or internodes of a plant stem.

#### Additional challenges in the application of anatomical axes

Although the designation of the primary organism axes may appear straightforward, pronounced developmental and evolutionary changes in organ presence, morphology, and symmetry in many taxonomic groups have made these axes biologically difficult to interpret and thus made it correspondingly difficult to apply a standardized terminology. The evolutionary shift to pentaradial symmetry in the adults of extant echinoderms, starfish, brittlestars, sea urchins, sand dollars, and crinoids is one of the most spectacular examples. All echinoderm larvae are bilaterally symmetrical, but upon metamorphosis, little or no trace of the larval AP axis remains in the pentaradial adult [24]. Whether there are five AP axes, one central AP axis, or none at all is still under molecular and genetic investigation. Similarly, the bilaterally symmetrical swimming larvae of tunicates, with their characteristic chordate features including pharyngeal arches and a post-anal tail, metamorphose into sedentary sac-like adults with no apparent remnant of an AP axis. The tapeworm lacks a clear AP axis: adults lack a digestive tract (no mouth or anus) and neither end contains a concentration of neurons that might be considered cephalic. Although the end with the holdfast organ (scolex) is commonly considered “anterior”, evidence including the manner of development of new segments and the positioning of testes relative to ovaries within segments points to the opposite conclusion [15]. Biologically meaningful application of anatomical position terms requires further molecular and genetic understanding of the development of these taxa.

Within plants, axis specification across species is fairly straightforward because of the association between axes and developmental patterns (i.e., the ‘apical-basal axis relative to direction of growth’ is associated with apical growth and the ‘medial-radial axis’ is associated with radial growth). Nonetheless, unusual developmental patterns, such as the adaxialization of cylindrical leaves (described above under “Axes of organism parts”) can obscure the normal axes used to describe plant structures. Thus, it is the precise specification of spatial terminology that allows for logical comparisons among forms that deviate from the norm.

### Anatomical planes and sections

Anatomical investigation is frequently based on histological sections (i.e., anatomical planes) to support a better understanding of three-dimensional structure. For example, long before the days of computerized image reconstruction, anatomists leveraged “coronal”, “horizontal”, “sagittal”, and “parasagittal” tissue sections to support inferred three-dimensional representation of anatomical entities within an animal. These are evident in numerous landmark atlases such as “The Rat Brain in Stereotaxic Coordinates” by Paxinos [25] and Kaufman’s “The Atlas of Mouse Development” [26]. Even in more modern digital approaches, reconstruction can happen only if the two-dimensional axes are accurately specified and registered (for examples, see [27]).

**Figure 5:**
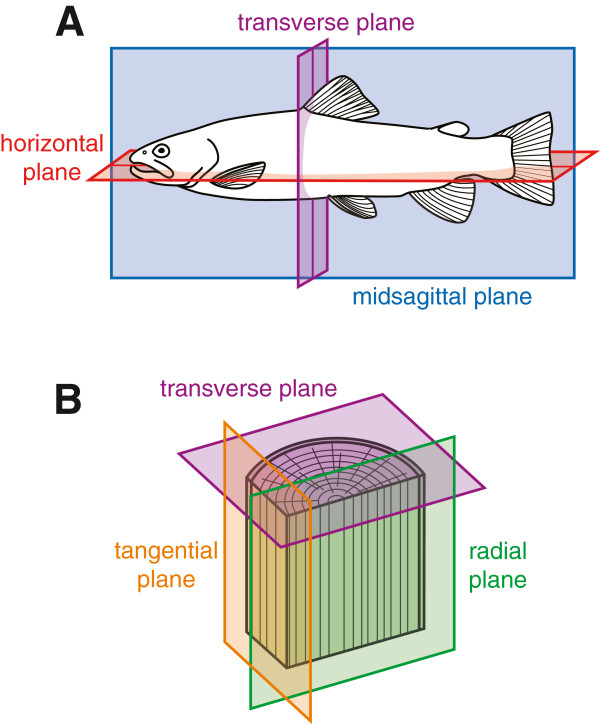
**Anatomical planes in BSPO. A)** The three anatomical planes used to describe bilaterally symmetrical organisms are ‘midsagittal plane’ (blue), ‘horizontal plane’ (red), and ‘transverse plane’ (purple). **B)** Anatomical planes used to describe wood (secondary xylem) anatomy. A ‘transverse plane’ (purple), or cross-section, is perpendicular to the ‘apical-basal axis relative to direction of growth’ in an axial organ or to a ‘proximal-distal axis’ in a lateral organ. A ‘radial plane’ (green) follows the two dimensions specified by an ‘apical-basal axis relative to direction of growth’ and a ‘medial-lateral axis’. A ‘tangential plane’ (orange) is perpendicular to a radial plane.

Anatomical planes (Figure 5) are defined as perpendicular or parallel to an axis in BSPO. For example, ‘sagittal plane’ is defined as “Anatomical plane that divides a bilateral body into left and right parts, not necessarily of even size” and has relationships *orthogonal_to* ‘left-right axis’ (Figure 2) and *parallel_to* ‘anterior-posterior axis’ and ‘dorsal-ventral axis’. The use of BSPO can aid integration and error-checking of section-based views through coordinates related to BSPO axes, based on the logic within the ontology. For example, if a histological feature is annotated to a particular structure that has in turn been declared to be located on the left side of the organism, a right parasagittal section should not include such a structure.

Traditional plant anatomy refers to three planes: ‘transverse plane’ (or “cross-section”), ‘radial plane’ , and ‘tangential plane’ (Figure 5B). The ‘transverse plane’ is used for plant parts that are both round or flattened in cross-section, such as stems or leaves, whereas ‘radial plane’ and ‘tangential plane’ are generally used only with structures that are roughly round in cross-section. All three planes are essential for the characterization and identification of wood (secondary xylem found generally in plant axes such as stems and roots), as woody tissues appear different in each plane [28].

The Foundational Model of Anatomy (FMA) ontology [29] for humans has an extensive classification of planes. These include ‘horizontal anatomical plane’ , ‘Frankfurt plane’ , and ‘thoraco-abdominal plane’. The FMA uses these planes to demarcate the boundaries of organism subdivisions such as the thorax. We cross-reference FMA classes where they exist in the representation of planes in BSPO but focus on planes that are widely applicable across organisms.

In addition to the relations along anatomical axes described above, we specify a number of relations relative to anatomical planes. For example, relative to the sagittal plane, *ipsilateral_to* holds between two structures on the same side of an organism; *contralateral_to* holds between two structures on the opposite sides of an organism. In contrast to the standard axial relations, these are not transitive, but they do hold the characteristic of being symmetric. If x is on the same side as y, then it must be the case that y is on the same side as x.

### Anatomical topology: regions, sides, and margins

Unlike axes and planes that are “immaterial”, the anatomical topology classes in BSPO refer to the material regions, sides, and margins of anatomical structures. These are labeled and defined in BSPO relative to the axis classes. For example, subtypes of ‘anatomical region’ (Figure 2) include ‘anterior region’ , ‘dorsal margin’ , and ‘posterior side’. These classes can be used to spatially define anatomical structures relative to an axis of the whole organism. Variation in the topology of homologous structures across species is informative for phylogenetic inference. Examples include differences in surface features, such as the textured or smooth surface of cranial bones in catfishes [30], differences in the margins of skeletal elements, such as the dorsal margin of the ilium in amniotes [31], and differences in the adaxial and abaxial regions of a leaf (Figure 4A).

Anatomical sides are defined with the non-transitive subproperties of *part_of* that specify which side of a bisecting plane a structure is part of. Where these reference the axes of the whole organism, they can apply to a side of either the whole organism or its substructures. For example, *in_left_side_of* can apply to the position of the heart relative to the whole organism, or apply to part of the heart, such as its left side. Where the referenced side only applies to some part of an organism, so do the relations. For example, in the long bones of limbs that have proximal and distal sides, the ‘proximal epiphysis of the femur’ can be defined as an ‘epiphysis’ that is *in_proximal_side_of* the ‘femur’. We also define property chains to propagate information about sides down the partonomy, so that, for example, if X *part_of* Y and Y *in_left_side_of* ‘heart’ then a reasoner can infer that X *in_left_side_of* the ‘heart’.

Some structures are not completely on one side or the other of a bisecting organismal plane but instead cross it. For example, the heart may asymmetrically span the midsagittal plane of an animal. For such cases, we define the relation: *intersects_midsagittal_plane_of*. This relation applies to midline structures such as the single unpaired nostril of the hagfish, which is positioned along the midline of its head (‘median external naris’ EquivalentTo ‘external naris’ and *intersects_midsaggital_plane_of* some ‘head’). This relation does not imply that the structure is unpaired, although this may often be the case. Structures to which the *intersects_midsaggital_plane_of* does not apply stand in a *in_lateral_side*_*of* relation to the whole. For example, in most vertebrates, the naris (nostril) is bilaterally paired, and it is thus declared in UBERON (the cross-species metazoan Uber Anatomy Ontology) [32,33] as being *in_lateral_side_of* a head. This relation does not imply, however, that the structure is paired. To indicate whether a structure is paired or unpaired, classes such as ‘bilateral’ from the Phenotype and Trait Ontology (PATO) [34] can be used, although further work needs to be done to connect these PATO classes to BSPO.

### Anatomical gradients

Anatomical gradients are defined in BSPO as “Material anatomical entity defined by change in the value of some quantity per unit of distance across some spatial axis.” Note that these classes are defined as structures whereby the differentiating characteristic is the distribution of some factor across a gradient. For example, Sonic hedgehog (Shh) is expressed in a posterior to anterior gradient in the developing limb buds of vertebrates. The concentration of Shh is interpreted by the cells and influences the phenotypic outcome of digit morphology according to the gradient [21]. An anatomical gradient can also be applicable to the whole organism, such as in the case of early anterior specification by *bicoid*, a maternal effect RNA that is translated in the fertilized egg and was discovered in *Drosophila melanogaster* in the 1980s (see [35] for review). Anatomical gradient subclasses for some of the primary organismal axes are included in BSPO, for example, ‘anterior-posterior gradient’ , which could be used to indicate the presence of the *bicoid* morphogen in the example above.

### BSPO and interoperability with other ontologies

The classes and relations in BSPO uniquely represent the spatial aspects of anatomical entities and can be used to create class expressions to enable spatial reasoning. UBERON simplifies the specification of spatial patterns in taxon-specific anatomy ontologies by doing this, e.g., UBERON:‘forelimb’ BSPO:*anterior_to* some UBERON:‘hindlimb’. Thus use of BSPO in UBERON can be leveraged to infer spatial relations by new or existing anatomy ontologies without those relationships. For example, ‘forelimb’ and ‘hindlimb’ in the Xenopus Anatomy Ontology (XAO) [36] reference the UBERON classes for ‘forelimb’ and ‘hindlimb’ , and therefore it can be inferred that a XAO:‘forelimb’ is *anterior_to* some XAO:‘hindlimb’.

Pre-composition using BSPO classes can enhance the definitions of some classes in anatomy ontologies that refer to the spatial aspects of structures. For example, the Plant Ontology [37,38], a unified vocabulary for all green plants contains a class for ‘phyllome base’ that is defined as “The basal part of a phyllome, where it attaches to a shoot axis.” Currently, only the relationship *part_of ‘*phyllome’ is specified in the ontology, but a more precise logical definition could be created by specifying that a ‘phyllome base’ is a BSPO ‘proximal region’ that is *part_of* a ‘phyllome’. The Gene Ontology (GO) also contains classes that could be defined in terms of BSPO classes and relations. For example, GO:‘AP axis specification’ is defined as “The establishment, maintenance and elaboration of the anterior-posterior axis. The anterior-posterior axis is defined by a line that runs from the head or mouth”. This class could also be formally related to BSPO: ‘anterior-posterior axis’.

Post-composition allows one to create classes that are more granular than those available in an anatomy ontology while avoiding the complexity and potential unwieldiness to the ontology that may result from pre-composing very specific classes [39]. Thus post-composing classes for the regions, margins, and surfaces of structures needed for annotation avoids creating a great number of pre-composed classes. BSPO is used by the Phenoscape project (Phenoscape.org; [40,41]) to create post-compositions for the annotation of morphological variation within and among vertebrate species resulting from evolution. For example, the posterior location of a bony projection on the cleithrum^b^ (a shoulder girdle bone) is represented by combining the following anatomical and spatial classes: ‘anatomical projection’ *part_of* some BSPO:‘posterior region’ and *part_of* some ‘cleithrum’. Spatial classes are also used to specify the region of a structure that varies in some quality; for example, BSPO:‘anterior margin’ *part_of* some ‘scapula’ is annotated as ‘concave’ or ‘straight’ using quality classes from PATO. BSPO is also used in post-composition for the annotation of gene expression in ZFIN (zfin.org; [42]) and used to formally represent taxonomic species descriptions for wasps [43].

PATO is an ontology of biological qualities that contains a number of relational qualities representing spatial concepts. Formally there is a difference between these spatial qualities and BSPO relations: PATO relational qualities are classes (e.g., ‘dorsal to’) rather than relations as in BSPO (e.g., *dorsal_to*). This difference manifests itself in concrete ways when modeling the world using languages such as the Web Ontology Language (OWL). For example, “A is dorsal to B” (where A and B are instances) is asserted as a simple triple < A *dorsal_to* B>. However, to refer to this “dorsality” relationship (e.g., to say that A is more dorsal to B than it is to C; or that this dorsality is caused by some genetic alteration), the relationship must be turned into an individual, i.e., a relational quality (reified relation). These spatial quality classes in PATO could be pre-composed with the relevant BSPO class. For example, ‘dorsalized’ is defined as a bearer's gross morphology containing only what are normally dorsal structures. This class could be formally defined by relating it to the BSPO class ‘dorsal region’.

### Use of BSPO for text mining

BSPO is useful at different levels for the natural language processing of morphological descriptions. For text mining software such as CharaParser [44], which is being developed to assist biocurators in annotating anatomical phenotypes, BSPO can be used at the lexical level as a dictionary for identifying spatial classes in free text descriptions. This most basic usage of the ontology makes more complex uses possible.

After spatial classes are identified at the syntactic level, BSPO is used to post-compose anatomical entities when pre-composed classes from an anatomy ontology, such as UBERON, are not available. For example, for the phrase “anterior margin of maxilla”, CharaParser would propose the expression BSPO:‘anterior margin’ and *part_of* some UBERON:‘maxilla’ after it failed to find term variations such as ‘anterior margin of maxilla’ , ‘maxilla anterior margin’ , or ‘maxillary anterior margin’ in UBERON. Phrases such as “anterior process of the maxilla” are handled similarly in that post-composition is considered only when pre-composed classes/components are not found in ontologies. In this case, CharaParser would propose the post-composition: UBERON: ‘anatomical projection’ (synonym: “process”) and *part_of* (BSPO: ‘anterior region’ and *part_of* UBERON: ‘maxilla’), along with other possible proposals.

Sometimes additional domain knowledge is needed to annotate a phenotype that is not obviously spatially related. For example, the semantics of the phenotype “clavicle blades articulate” is built on the knowledge that clavicle blades are bilaterally paired structures. The BSPO *in_left_side_of* and *in_right_side_of* relations (children of the BSPO relation *in_lateral_side_of*) can be used to explicitly define this type of structure. This makes it possible for CharaParser to use the ELK reasoner [45] to find all structures that are bilaterally paired in UBERON by obtaining the union of (BSPO:*in_lateral_side_of* some Thing) and (*part_of* some BSPO:*in_lateral_side_of* some Thing). When CharaParser processes qualities that are in the relation_slim of PATO, such as ‘articulated with’ , it will understand that two entities are expected and then look into a list of bilaterally paired structures for possible matches for the two entities (i.e., ‘clavicle blade’ and (*in_left_side_of* some ‘multi-cellular organism’) *and ‘*clavicle blade’ and (*in_right_side_of* some ‘multi-cellular organism’)). Note that non-bilaterally paired structures can also use PATO relational qualities (e.g., ‘frontal’ PATO: ‘articulated with’ ‘parietal’).

### Towards formalization of BSPO relations

The BSPO is represented in OWL, which provides a limited number of constructs for characterizing relationship types. We make use of characteristics such as transitivity, superproperties, and domain/range constraints to allow for limited forms of reasoning. For example, if A is anterior to B, and B is anterior to C, then the transitivity characteristic of *anterior_to* entails that A is anterior to C. Similarly, we can also trivially infer that C is *posterior_to* A, using inverse axioms. Other more sophisticated forms of reasoning are not possible at this time. For example, the *orthogonal_to* relation has domain and range constraints (it holds between an axis and a plane), but it has no definitional axioms that capture the textual definition of crossing a plane at a right angle. Spatial extensions to OWL would be required to rigorously capture this meaning, but it is not clear what the use case for these advanced types of reasoning would be. One possibility would be the integration of classic description logic queries with geometric 3D model or anatomical atlas data. For example, asking for all genes expressed in epithelial cells dorsal to a plane formed by bisecting a particular organ. One possibility is to extend OWL using custom datatypes – this is possible using a system such as OWL-Eu [46]. For many practical scenarios, it may be sufficient to encode the logic of the relation directly into the query engine. This is an area that would require further exploration.

## Conclusions

The BSPO supports unambiguous usage of positional terminology in the context of anatomical data and in the building of anatomy ontologies. BSPO also serves as a source of classes and relations for post-composition of anatomical entities, a requirement for the representation of morphological variation within and among species. To aid in its use, we include textual information indicating the ‘taxon-appropriateness’ of different classes and relationships in BSPO. In the future, we will also include taxon constraints [47] and add additional constraints encoded as OWL axioms.

The BSPO provides an ontological representation of anatomical position classes that can be used for spatial reasoning. For example, queries can be enabled to find structures that are proximal to one another, or to compare levels of phenotypic variation in dorsal vs. ventral regions. Particularly in light of the high level of conservation in gene pathways underlying these axes across species (e.g., BMP gradients in dorsal-ventral patterning), the BSPO is critical to enable interesting queries across phenotypes at different anatomical positions.

## Methods

BSPO contains classes and relations (*object properties* in OWL) for the representation of anatomical axes, gradients, regions, planes, sides and surfaces (Figure 2). Spatial classes are classified along a single subclass hierarchy with upper level classes (e.g., ‘material anatomical entity’ , ‘immaterial anatomical entity’) imported from CARO. Coordination of classes with a new CARO release is ongoing, and we anticipate making a coincident new release of both ontologies soon. Some relations (e.g., *part_of*) used in the BSPO are defined in the Relations Ontology [48] and more specific relations (e.g., *posterior_to*) are exclusively defined in BSPO. The specific relations in the BSPO currently lack higher-level parents in the Relations Ontology. Some relations in BSPO are used to relate anatomical region classes to those of anatomical axis, such as ‘anterior side’ which has a *starts_axis* relationship to ‘anterior-posterior axis’ (Figure 2). Other commonly used relations in BSPO include *overlaps* (e.g., ‘anterior region’ *overlaps* ‘anterior side’), and *surface_of* (e.g., ‘anterior surface’ is a *surface_of* ‘anterior side’). Note that we define relations textually but we are unaware of a way to create a complete formal definition using OWL, which has limited capabilities for reasoning with relations.

The original version of BSPO was derived from the FlyBase annotation qualifier section of the FlyBase controlled vocabulary (FBql). We retain cross-references to the original FBcv classes. An editor’s version of the ontology is maintained in OBO format and edited using OBO-Edit [49]. Public releases are made in both OBO and OWL versions of the ontology. Requests for ontology changes and additions can be made on the term request tracker (https://code.google.com/p/biological-spatial-ontology/issues/list).

## Endnotes

^a^We follow the Open Biological and Biomedical Ontologies (OBO Foundry) convention of referring to concepts as “classes” and the relationships between classes as “relations”, as opposed to “classes” and “object properties” in the Web Ontology Language (OWL). Throughout the text, classes are denoted in single quotes and relations in italics.

^b^The cleithrum is a bony element represented in the Teleost Anatomy Ontology [50] in relation to mode of skeletal development according to the Vertebrate Skeletal Anatomy Ontology [51]. These ontologies have been merged into the comprehensive Uberon anatomy ontology [33].

## Competing interests

The authors declare that they have no competing interests.

## Authors’ contributions

All authors contributed to the ontology and the manuscript. WD, MAH, PM, and RLW developed the figures. All authors read and approved the final manuscript.
